# Standardizing Disaster Health Data Visualization Using Fast Healthcare Interoperability Resources in Indonesia: Dashboard Development and Technical Evaluation

**DOI:** 10.2196/98818

**Published:** 2026-10-02

**Authors:** Hiro Putra Faisal, Masaharu Nakayama

**Affiliations:** 1 Department of Physiology, Faculty of Medicine, Syarif Hidayatullah State Islamic University Jakarta, Tangerang Selatan, Banten, Indonesia; 2 Department of Medical Informatics, Graduate School of Medicine, Tohoku University, Seiryo-machi, Aoba-ku, Sendai, Miyagi, 980-8574, Japan, 81 22-717-7572, 81 22-717-7505

**Keywords:** World Health Organization Emergency Medical Teams Minimum Data Set, WHO EMT MDS, Fast Healthcare Interoperability Resources, FHIR, disaster, dashboard, data visualization

## Abstract

**Background:**

Health data management during disasters enables responders to assess the needs of survivors, efficiently allocate resources, and monitor survivors’ health conditions. However, government agencies need to interpret health data in real time during disasters because the volume and complexity of such data can hinder timely decision-making.

**Objective:**

In this study, we aimed to develop a visual interface for Fast Healthcare Interoperability Resources (FHIR)–based data from various sources, using the daily report form of the World Health Organization (WHO) Emergency Medical Teams Minimum Data Set (EMT MDS) as a template and ensuring compliance with the SATUSEHAT platform.

**Methods:**

The proposed solution involves creating a dashboard application within a web-based system to visualize health data using FHIR, leveraging the WHO EMT MDS disaster profile. The interface includes Overview, Location, and Demography pages that display information in tables, graphs, and maps. This application was developed using JavaScript and React. The dataset consisted of 13,300 synthetic patient records generated using Synthea, subsequently modified with GPT-5, and stored on the Health Level 7 (HL7) Application Programming Interface (HAPI) FHIR server.

**Results:**

We developed a dashboard application based on the WHO EMT MDS Daily Reporting Form comprising 3 main pages: Overview, Demography, and Location. The Demography page contains several sections, including the total number of patients, health conditions, outcomes, relationships, and protection. The Location page lists all health care facilities, along with their corresponding locations, on a map. Each Location page displays detailed information regarding the organization and demographics of the respective location.

**Conclusions:**

The dashboard application demonstrates that health data collected in accordance with FHIR standards can be integrated and presented in a form suitable for disaster response. This dashboard has the potential to support disaster management agencies by enabling real-time, data-driven decision-making during disaster response.

## Introduction

Indonesia is among the most disaster-prone countries due to its geographical, geological, and climatological conditions [1,2]. The consequences of these risks were evident in several disasters in recent years, including earthquakes, volcanic eruptions, and, more recently, floods and landslides in Sumatra in late 2025 that displaced more than 390,000 people across 3 provinces [3]. Such large-scale, recurring events underscore the critical need for robust health information systems to support timely, coordinated disaster response.

Frontline health services during disasters are delivered primarily through *Puskesmas* (community health centers), which operate at the subdistrict level and serve as the first point of contact for affected populations [4,5]. Each *Puskesmas* typically covers a catchment area of approximately 30,000 residents [6], making it a key operational unit for aggregating information and achieving rapid situational awareness during disasters. However, disaster response often involves multiple agencies and teams, and health data are frequently fragmented across different reporting channels, limiting operational decision-making and coordination [7-9].

To address data fragmentation and improve coordination, the World Health Organization (WHO) introduced the Emergency Medical Teams Minimum Data Set (EMT MDS) in 2017 as a standardized framework for systematic data collection and reporting during emergencies [10]. In parallel, Indonesia has initiated nationwide health data interoperability through the Ministry of Health (MoH) SATUSEHAT platform, which adopts Fast Healthcare Interoperability Resources (FHIR) as the technical standard for exchanging health data among electronic medical record systems [11,12]. These developments provide an opportunity to harmonize disaster health reporting with interoperable data infrastructures.

Our previous work proposed a FHIR-based profile to represent key elements of WHO disaster reporting and to facilitate interoperability in disaster contexts [13]. In this study, we aimed to develop a web-based dashboard that aggregates FHIR data and visualizes WHO EMT MDS–structured disaster response data to support timely situational awareness and coordination among responders and coordinating authorities, aligned with Indonesia’s FHIR-based data exchange approach.

## Methods

### Profile

We used the EMT MDS disaster profile mapped to the FHIR standard [13]. The profile contains an extension applied to the FHIR “Condition” resource to capture the relationship between a survivor’s health condition and the disaster event (directly related, indirectly related, or not related). The profile includes “StructureDefinition,” “ValueSet,” “CodeSystem,” and “ImplementationGuide” artifacts to ensure interoperability. All resources were represented as Health Level 7 (HL7) FHIR R4 and validated against the disaster profile “ImplementationGuide” on the Simplifier.net (Firely) platform [14], confirming conformance with required elements and bindings.

### Architecture and Interface Design

The dashboard application developed in this study was a web-based system structured around the EMT MDS Daily Reporting Form. It comprised 3 main sections: Overview, Location, and Demography. The front-end interface design was implemented using React (version 18.3.1; React Foundation), a JavaScript (Oracle Corporation) library known for its component-based architecture and ease of integration with dynamic datasets [15].

The Overview page displays aggregated daily visits over a 1-week period, while overall trends can be assessed across the full observation period. The Location page displays the geographic location and identity of the medical aid posts along with the corresponding emergency medical personnel. Geographic visualization was implemented using the Leaflet JavaScript library, which renders an interactive map using geocode data extracted from the FHIR “Location” resource [16]. The Demography page presents detailed demographic and clinical profiles of patients presenting at all medical aid posts. The attributes included distributions by age and sex, health conditions, procedures performed, relation to the disaster, and protection concerns, similar to the daily reporting forms in the EMT MDS.

The backend was developed using the NestJS framework (version 11.0.1), a server-side environment built on Node.js (version 22.2.0; OpenJS Foundation) [17]. This framework managed the application logic, handled requests from the front end, and communicated with the database and the FHIR server. We used the MySQL (version 8.0.35; Oracle Corporation) database to store nonclinical and administrative information. We used simple authentication to ensure secure access for users. System administrators manually generated and distributed user credentials.

### Data Extraction and Aggregation

Daily visit volumes were calculated by counting the number of “Encounter” resources per day. Patient counts were stratified into 3 categories (“Male,” “Female not pregnant,” and “Female pregnant”). Sex was obtained from the “Patient.gender” element, and pregnancy status was determined using pregnancy-related entries in the “Condition” resource for female patients. Health care facility lists were derived from the “Location” and “Organization” resources, and markers were plotted from the “Location.position” element and displayed on an interactive map using Leaflet (version 1.9.4) [16].

### Data Generation and Preparation

We generated a synthetic dataset to simulate disaster conditions in Indonesia and evaluate our application using Synthea (The MITRE Corporation), an open-source synthetic patient generator, as the core dataset in HL7 FHIR format [18]. We chose Synthea because its structure closely matches EMT MDS elements in HL7 FHIR format, ensuring high compatibility with the SATUSEHAT platform, as shown in Table 1.

To contextualize the dataset for the Indonesian health care system and disaster setting, we leveraged OpenAI’s GPT-5 [19], a publicly available large language model (LLM), to modify and enrich the baseline dataset by adjusting disease-related conditions and procedure resources to reflect disaster-related patterns and restructuring health care facilities to reflect health care settings in Indonesia. The “Encounter” resources were assigned to district hospitals, subdistrict *Puskesmas*, auxiliary health centers, and clinics as the primary health care settings. Additionally, we modified encounter time stamps to simulate temporal patterns consistent with disaster events, including sudden surges in patient visits during acute disaster phases and gradual increases during the postdisaster recovery period.

The test dataset comprised location lists and patient records, with ages ranging from 0 to 100 years and encounter events occurring over 1 month. Finally, we added the relationship status to the Condition resource for each patient in the JSON files to indicate whether the condition was related to the disaster, as in the daily reporting form, as the files lacked an extension for this purpose.

**Table 1.** Mapping of Emergency Medical Teams Minimum Data Set (EMT MDS) data elements from Fast Healthcare Interoperability Resources (FHIR).

EMT MDS elements	Resource elements	Description
Age	Patient.birthDate	Patient’s age is calculated from the patient’s birth date and the current date.
Sex	Patient.gender	—^a^
Pregnancy status	Condition.code	—
Health care visit	Encounter.period	Data on the health care visit are obtained from the start time of the encounter.
Health condition	Condition.code	—
Procedure	Procedure.code	—
Relation	Condition.extension	The extension is derived from the disaster profile.
Protection	Condition.code	—
Organization name	Organization.identifier	—
Location name	Location.name	—
Location address	Location.address	—
Location phone	Location.telecom	—
Location geotag	Location.position	The geotag is obtained from the longitude and latitude of the position element. These data are integrated into the map using Leaflet JavaScript library.

a Self-explanatory.

### Server Deployment and Data Integration

The generated dataset was stored on the HL7 Application Programming Interface (HAPI) FHIR Server, an open-source implementation of the FHIR standard that supports the storage, retrieval, and interoperability of clinical data using Representational State Transfer (RESTful) protocols. The HAPI FHIR server is widely adopted in health care research for its comprehensive support for all FHIR resources and compliance with HL7 standards [20]. The dashboard backend was containerized and deployed using Docker (version 4.58.1; Docker Inc) [21] on macOS (Apple Inc), with HAPI FHIR R4 (version 8.0.0; Smile Digital Health) and PostgreSQL (version 17.5; PostgreSQL Global Development Group) as the relational database backend for persistent FHIR storage.

Communication between the HAPI FHIR server and the dashboard application was established via a RESTful API, enabling real-time retrieval and integration of structured health data into the application. In addition, we stored the user authentication data in a dedicated database. This database operates independently of the FHIR store and supports the simple password-based authentication mechanism used in the dashboard. The detailed process is illustrated in Figure 1.

**Figure 1.**
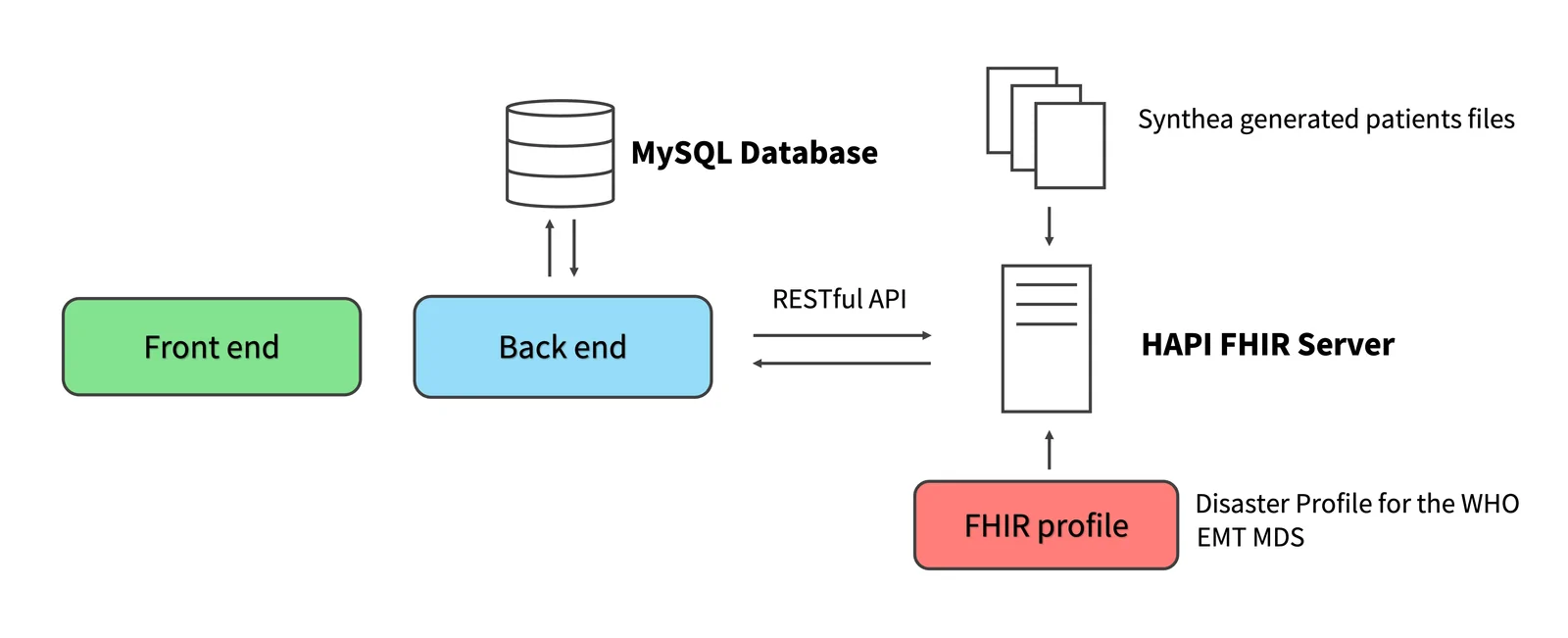
System architecture of the dashboard application. EMT MDS: Emergency Medical Teams Minimum Data Set; FHIR: Fast Healthcare Interoperability Resources; HAPI: HL7 Application Programming Interface; WHO: World Health Organization.

### Performance Evaluation

System performance was evaluated in a controlled local test environment. Dashboard metrics were computed over a rolling 7-day window. Encounter counts represented the total number of “Encounter” resources within the 7-day period, while patient counts represented the number of unique patients identified from “Encounter.subject” references within the same window.

Initial dashboard load time was measured using Google Chrome DevTools (Alphabet Inc) [22], with the total page load duration recorded as the “Finish” time after a full-page reload with browser caching disabled. Core API response times were measured as the “Duration” for each backend API request in the DevTools Timing panel. Each performance metric was measured 30 times under conditions without throttling. We report the median and IQR (25th-75th percentiles) across the 30 repeated measurements.

### Ethical Considerations

This study did not involve human participants, actual patient data, or human-derived biological samples or information, as all data were synthetic, generated using Synthea, and then adapted through AI-assisted transformation. According to Tohoku University’s policy on research involving human participants, which considers activities conducted on human research participants or using human-derived samples or information as requiring ethics review [23], this study does not qualify as research requiring such review. Accordingly, ethics board review was not sought, no case or application number is applicable, and informed consent was not applicable.

## Results

### Overview

The dashboard application successfully rendered data stored on the FHIR server, consisting of 3 main pages: Overview, Demography, and Location. To demonstrate the application workflow, we generated a dataset consisting of 13,300 synthetic patient records using GPT-5. The dataset included 31,920 encounters across 2 district hospitals, 4 subdistrict *Puskesmas*, 18 auxiliary health centers, and 10 clinics. A total of 17 condition types (33,810 records) and 5 procedure types (960 records) were included. Table 2 summarizes patient demographics. Figure 2 shows the daily visit volume calculated from encounter dates, with a midmonth surge from approximately 1000 visits per day to a peak of more than 2000 visits per day during the observation period.

**Table 2.** Patient demographics from the dataset (N=13,300).

Characteristics	Patients, n (%)
Age <1 year	
Male	161 (1.21)
Female	163 (1.23)
Age 1-4 years	
Male	310 (2.33)
Female	323 (2.43)
Age 5-17 years	
Male	1036 (7.79)
Female not pregnant	1047 (7.87)
Female pregnant	13 (0.1)
Age 18-64 years	
Male	3677 (27.65)
Female not pregnant	3766 (28.32)
Female pregnant	253 (1.9)
Age ≥65 years	
Male	1198 (9.01)
Female not pregnant	1353 (10.17)
Female pregnant	0 (0)

**Figure 2.**
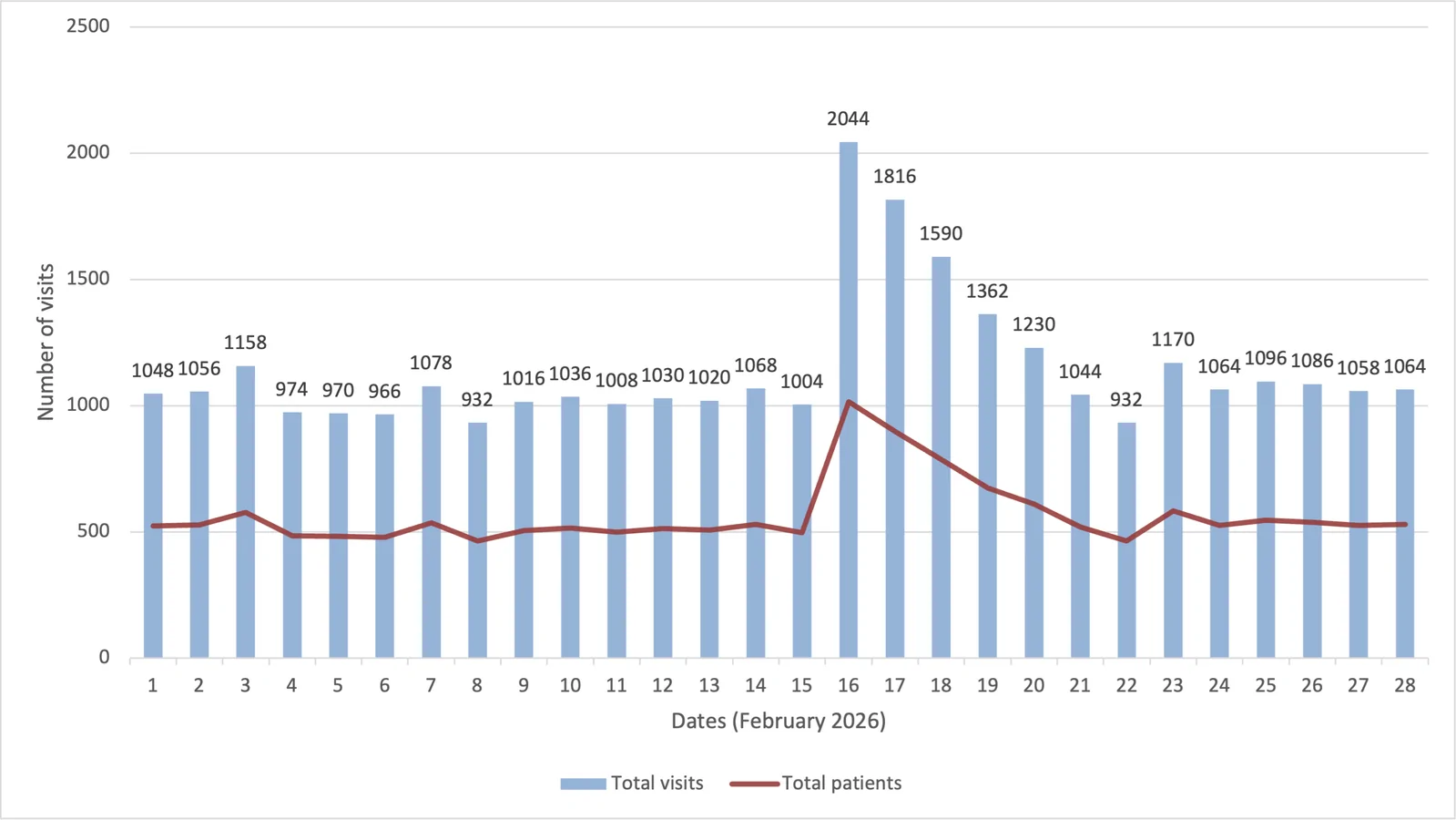
Total number of visits per day.

### Dashboard

The Overview page (Figure 3A) presents aggregated data from all health care facilities within the region. The weekly visit (encounter) display shows the total number of visits across all health care facilities as a bar chart. The patient summary chart presents counts by sex and pregnancy status (men, nonpregnant women, and pregnant women). The last section lists health care facilities and their corresponding organizations and locations.

**Figure 3.**
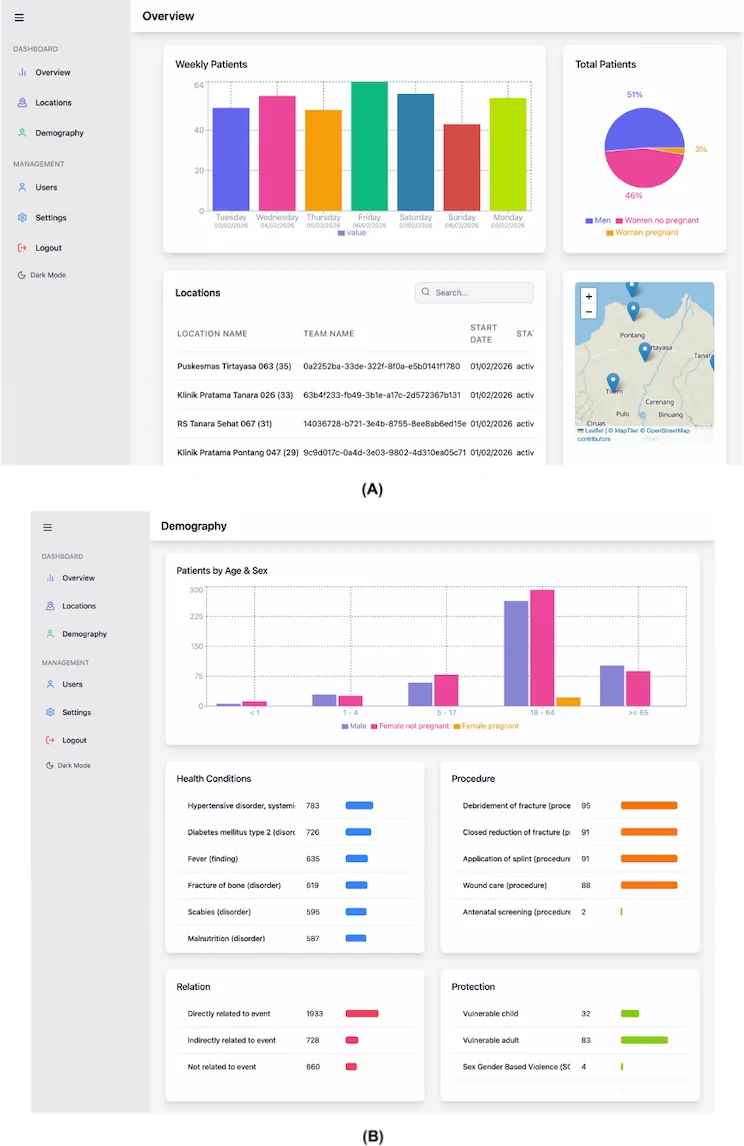
(A) The Overview page comprises data collected from all health care facilities within the region. Specifically, the page illustrates the total number of patients in 1 week, differentiated by sex and location of the health care facilities. (B) The Demography page illustrates the total number of patients from all health care facilities from the start date. Specifically, the page consists of sections for patients by age and sex, health conditions, procedures, outcomes, relations, and protection.

The Demography page (Figure 3B) compiles patient-level summaries across all health care facilities and presents multiple sections mapped to the WHO EMT MDS Daily Reporting Form. The top bar chart shows patient counts by age group and sex. The health conditions section lists conditions derived from the “Condition” resource. The procedure section lists procedures derived from the “Procedure” resource. The relation section indicates whether each condition is related to a disaster event based on the extension in the “Condition” resource. Finally, the Protection section summarizes counts of vulnerable and violence-related cases derived from the “Condition” resource.

The Location page (Figure 4) lists all active health care facilities and displays their locations on an interactive map using latitude and longitude information from the “Location.position” element. Each facility name links to a dedicated facility page that provides additional details, including the responsible organization and facility-level patient demographics. These views correspond to the EMT MDS daily reporting structure.

**Figure 4.**
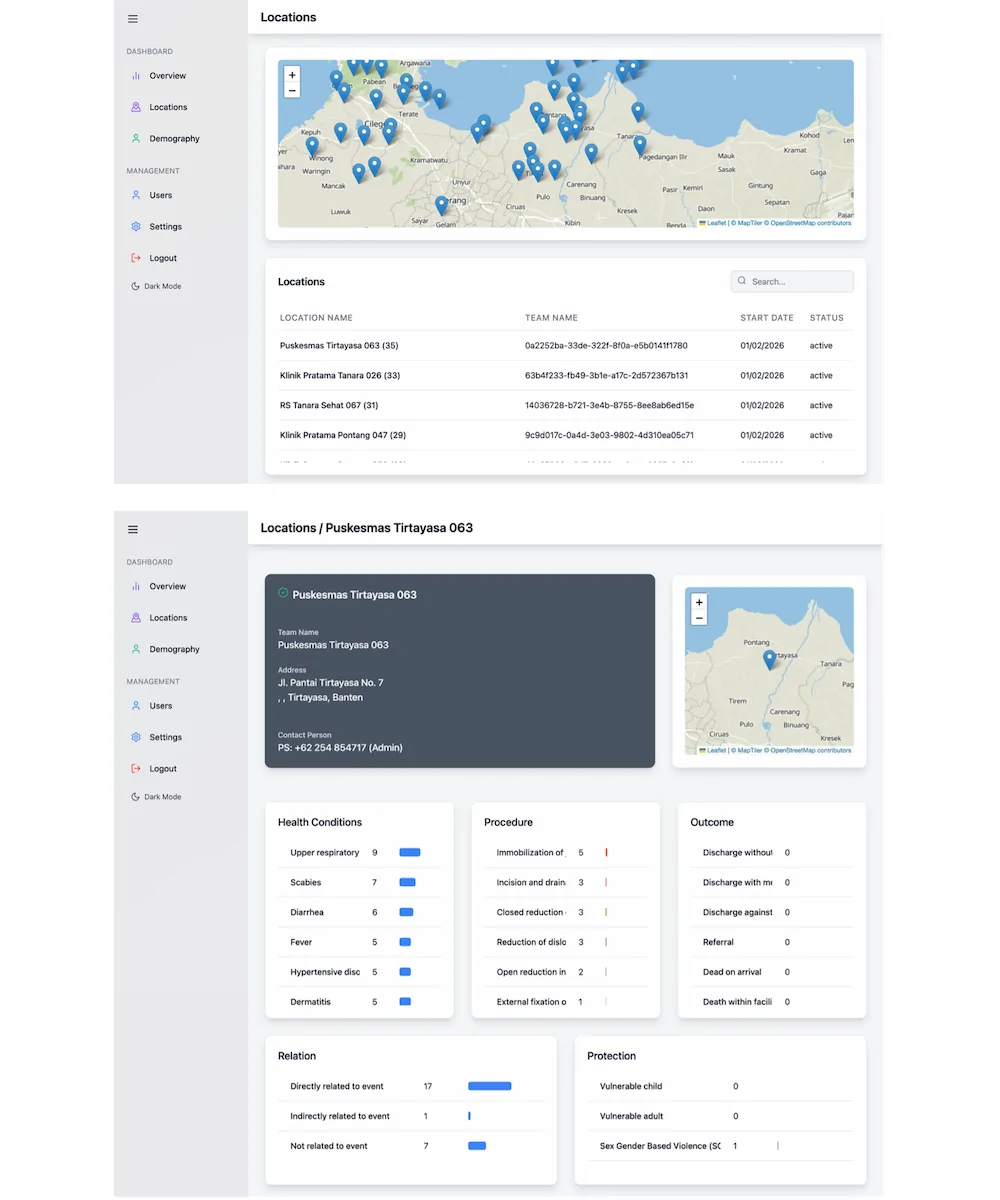
The Location page lists the health care facilities within the area, marked on the map. Each location is linked to a new page, providing detailed information about the health care facility.

With a 7-day rolling dataset comprising 3663 patients and 3811 encounters, the median initial dashboard load time was 5.36 (IQR 4.73-6.25) seconds. The median API response times were 1.86 (IQR 1.76-2.03) seconds for WeeklyPatients, 4.87 (IQR 4.56-5.62) seconds for PatientCount, and 2.68 (IQR 2.55-2.78) seconds for Top5Location, based on 30 repeated measurements per end point.

## Discussion

### Principal Findings

We developed a web-based dashboard that visualizes disaster response data structured according to the WHO EMT MDS Daily Reporting Form using FHIR. The dashboard displays 3 operational pages (Overview, Demography, and Location) that summarize patient demographics and facility-level information to support timely situational awareness and coordination. Given the limited availability of health-focused dashboards designed for the disaster response phase, our approach demonstrates how EMT MDS–aligned reporting can be operationalized on a FHIR-based interoperability infrastructure.

The WHO EMT MDS Daily Reporting Form provides a standardized structure to support Emergency Medical Team Coordination Cell (EMTCC) situational awareness and coordination among responding teams [24]. In Indonesia, the *Badan Nasional Penanggulangan Bencana* (BNPB; National Disaster Management Agency) coordinates disaster response at the national level and the *Badan Penanggulangan Bencana Daerah* (BPBD; Regional Disaster Management Agency) coordinates response at the regional level and both manage data flow during disasters. Disaster response requires dynamic, provisional data on the disaster itself, damage to facilities and infrastructure, and the impact of the disaster on affected populations [25]. In this workflow, our dashboard may reduce cognitive load by converting heterogeneous reports into interpretable summaries, serving as a reporting and visualization layer that aggregates facility-level data and presents summaries based on the EMT MDS to support coordination between field teams and coordinating bodies.

Figure 5 illustrates the roles of different organizations and government institutions and highlights how this visualization layer can support timely situational awareness. The primary intended users include EMTCC or Health Emergency Operation Center (HEOC) analysts and coordinating officers requiring cross-facility summaries during response operations.

**Figure 5.**
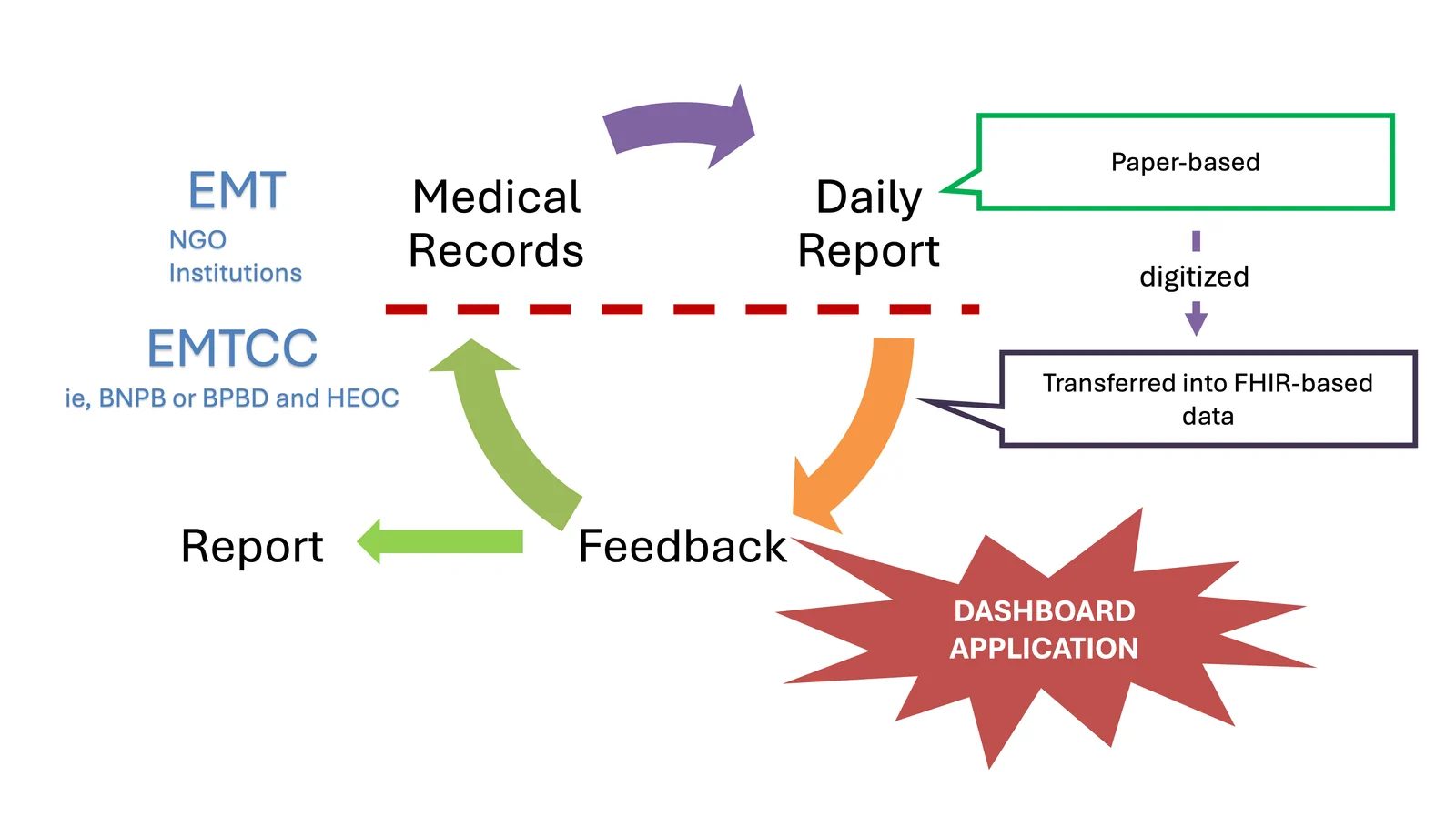
Data management flow during the disaster response phase. BNPB: *Badan Nasional Penanggulangan Bencana*; BPBD: *Badan Penanggulangan Bencana Daerah*; EMT: emergency medical team; EMTCC: Emergency Medical Team Coordination Cell; FHIR: Fast Healthcare Interoperability Resources; HEOC: Health Emergency Operation Center; NGO: nongovernmental organization.

During the response phase, the MoH activates the HEOC [26], integrating facilities, devices, trained resources, and information and communication technology systems, such as the *Sistem Penilaian Kapabilitas Kelembagaan* (SIPKK; Health Crisis Management Information System), to act as a control and collaboration center for monitoring and responding to health crises [27]. Consequently, the proposed dashboard application complements the HEOC monitoring function by reducing manual consolidation of data across heterogeneous reporting channels into standardized, near–real-time summaries of use and facility status.

However, the dashboard has not yet been tested under actual disaster conditions and is intended specifically for use in small- to medium-scale disasters, where communication infrastructure remains functional with minimal disruption. These include localized or slow-onset events in which physical damage to telecommunication towers is limited and the power supply remains available [28,29], both of which are needed for the HEOC to operate continuously during disasters. Potential deployment scenarios include urban flooding in Jakarta, where digital platforms exist but lack health monitoring and surveillance [30], and volcanic events such as the eruption of Mount Ruang, where the HEOC relies on digital surveillance in displacement centers [31]. Finally, prior research has also identified a need for an application supporting data input from primary health care centers with graphical reporting capabilities [32], which this dashboard addresses.

As shown in Figure 5, the dashboard’s outputs flow in 2 directions: feedback returned to emergency medical team (EMT) institutions to inform ongoing field-level data collection and consolidated reports disseminated to EMTCC or BNPB and government coordinating bodies for cross-facility decision-making. This bidirectional flow positions the dashboard as an active node that also supports 2-way communication between frontline teams and coordinating authorities.

### Dataset

Finding an ideal dataset that aligns with the WHO EMT MDS proved challenging. Among available FHIR-based datasets, we selected Synthea because it covers 12 of the 15 required FHIR resources listed in Table 3. As the default Synthea modules reflected the epidemiological profile of the US population, we used AI to adapt the dataset to simulate disaster scenarios common in Indonesia [33,34]. We adjusted the conditions and procedures to reflect disaster-related diseases, including trauma, waterborne diseases, infectious diseases, and noncommunicable diseases that affect therapy. This epidemiological alignment mitigates population and representation bias, ensuring the data reflect local disease prevalence and resource constraints [35,36]. The use of AI has been demonstrated to effectively produce realistic, contextually appropriate synthetic clinical data while maintaining structural consistency with FHIR standards [37,38].

**Table 3.** Comparison of Fast Healthcare Interoperability Resources (FHIR) between Synthea and World Health Organization Emergency Medical Teams Minimum Data Set (EMT MDS) data elements.

EMT MDS FHIR data elements	Synthea
Allergy intolerance	✓
Communication	
Condition	✓
Encounter	✓
Imaging study	✓
Immunization	✓
Location	✓
Medication request	✓
Medication statement	
Observation	✓
Organization	✓
Patient	✓
Practitioner	✓
Procedure	✓
Service request	

The synthetic dataset was sufficient for demonstrating application behavior under simulated conditions and for early-stage prototyping. The baseline dataset was refined using AI-assisted transformation to simulate disaster-specific scenarios, including increases in disaster-related conditions and a surge in health care use across multiple facilities. This allowed us to model temporal spikes in patient visits consistent with emergency events, which the application successfully visualized as distinct increases in daily encounters. This ability to show patterns within the application demonstrates the dashboard’s capacity to detect abnormal use patterns and support disaster situational awareness.

Although the AI-assisted synthetic dataset effectively demonstrated the dashboard’s technical capability to detect surges in patient visits, it does not fully capture the complexity and variability inherent in real disaster events. Real-world disaster data are typically characterized by incomplete or delayed reporting, inconsistent data quality across facilities, and clinical presentations shaped by comorbidities and context-specific factors that are difficult to simulate systematically. Furthermore, the synthetic scenarios used in this study reflect a generalized disaster pattern and may not capture heterogeneity across disaster types (eg, earthquakes, floods, and infectious disease outbreaks) or the operational obstacles responders face in the field. Thus, the results should be interpreted as a demonstration of technical feasibility rather than a validated representation of real disaster response dynamics.

### Relation, Outcome, and Protection

We integrated a relation extension validated on Simplifier.net to capture the relationship between a patient’s condition and disaster events. Meanwhile, we did not find Systematized Nomenclature of Medicine–Clinical Terms (SNOMED CT) codes for the protection section that directly matched the variables used in the EMT MDS. This finding can be attributed to the differences in design; the EMT MDS group variables simplify data entry and make it easier for the response team to complete during a disaster [39], whereas SNOMED CT provides highly granular clinical terminology [40,41]. Therefore, representing these concepts often requires using multiple codes. Table 4 presents relevant sample codes used in this study.

**Table 4.** List of contexts of the protection section in the World Health Organization Emergency Medical Teams Minimum Data Set (EMT MDS) and their relation to Systematized Nomenclature of Medicine–Clinical Terms (SNOMED CT) codes.

Descriptions	SNOMED CT code
Vulnerable child	
Child at increased risk of adverse outcome (finding)	138198004
Child at increased risk of neglect (finding)	704658004
Vulnerable child in family (situation)	160891002
Vulnerable adult	
Vulnerable adult (finding)	417430008
Adult safeguarding concern (finding)	766561000000109
SGBV^a^	
Sexual abuse (event)	213017001
At increased risk of domestic violence (finding)	707087005
Victim of sexual aggression (finding)	56890008
Non-SGBV	
Assault (event)	52684005
Victim of abuse	386702006

a SGBV: sexual gender–based violence.

### Performance Measurement

This research used the HAPI FHIR server as the data source populated with the synthetic dataset. The dashboard application retrieves data from this server via RESTful API calls and performs dashboard computations on the retrieved data, which are cached at the application layer to avoid redundant recomputation within an active session. This client-side caching reduces repeated queries during a session; however, the current architecture is not designed to operate autonomously when this connection is unavailable. In a real-world deployment connecting to SATUSEHAT, the dashboard would inherit a comparable connectivity dependency, and this study has not evaluated its resilience under network disruption or degraded infrastructure. However, this study did not establish whether these load and response times would remain acceptable in larger deployments involving substantially more facilities and patients.

On the basis of the results, the dashboard’s median initial load time (5.36, IQR 4.56-5.62 seconds) and median API response times (ranging from 1.86 to 4.87 seconds across evaluated end points, IQR 1.76-2.03) were obtained using a dataset intentionally scaled to reflect small- to medium-scale disaster scenarios, such as localized flooding or landslides. These results are broadly consistent with established human-computer interaction benchmarks for acceptable response times. The basic thresholds suggest that operations completed within 10 seconds still hold user attention, while responses under 2 seconds support an uninterrupted flow of thought [42]. Empirical work on user tolerance for web-based information retrieval similarly found that users begin noticing delays after about 2 seconds but do not abandon a task until delays exceed approximately 15 seconds [43]. The dashboard’s median query response times and initial load time fall within these tolerances, although the initial load exceeds the 2-second mark at which users first perceive a slowdown.

Comparable dashboard systems in the health and emergency domains report a wide range of performance depending on architecture and data model. District Health Information Software 2 (DHIS2), the health information system used by ministries of health in more than 80 countries, has documented dashboard item load times ranging from >0.1 seconds to <100 seconds when dashboards query large volumes of unaggregated tracker-level data directly [44]. These results show that data aggregation can determine dashboard responsiveness at scale. Two approaches may be considered to address this issue: cloning the server to provide greater control over data management or implementing additional backend filters to reduce query load. Future development should evaluate both approaches for scalability, implementation complexity, and compatibility with the existing server infrastructure before adopting a definitive solution.

### Authentication

The proposed dashboard application uses simple authentication to restrict access to authorized users and reduce unnecessary system requests. System administrators can adjust the data collection start date and add new users as needed.

### Future Plan

Future implementation plans target Indonesian disaster management, leveraging FHIR’s alignment with SATUSEHAT, Indonesia’s national FHIR-based platform for integrating electronic medical records, to ensure compatibility with the country’s digital health transformation and to strengthen the potential for large-scale adoption during disaster response. Future work will also include usability and field validation with EMT and EMTCC or HEOC personnel to assess the dashboard’s operational performance, extending beyond the technical concept demonstrated in this study.

Furthermore, displaying health data in near real time during disasters has considerable potential to enhance response capacity. For example, integrating AI-based predictive analysis can enable the early detection of potential disease outbreaks [39]. Such predictive capabilities enhance preparedness by allowing decision-makers to anticipate health needs, allocate resources more efficiently, and implement timely interventions. Ultimately, this approach could help mitigate adverse health outcomes and strengthen resilience during public health emergencies.

### Limitations

#### JavaScript Functions

Currently, the dashboard performs data calculations using JavaScript functions, thereby providing immediate results. However, challenges arise when processing large volumes of data. Consequently, a more robust solution would involve shifting the computational tasks to the backend, for example, by using SQL queries or a dedicated computation engine [45].

#### Synthetic Data

This research uses synthetic data because a disaster health dataset that complies with the FHIR standard does not currently exist, due to privacy restrictions on real patient records and the absence of a standardized national disaster health registry. We used Synthea as the base data generator and modified the dataset to reflect Indonesian disaster-related epidemiology, as the Synthea dataset is based on US demographic and clinical patterns and can be generated in the FHIR standard. This adaptation process became an important limitation because it has not undergone formal clinical validation by domain experts and should be addressed through expert review or real-world data in future work. As synthetic data of this type cannot fully capture the complexity and variability of real disaster scenarios, the results presented in this study should be interpreted as a demonstration of technical feasibility rather than a validated representation of real-world disaster response.

#### Authentication and Privacy

The dashboard developed in this study uses a simple authentication mechanism. However, broader deployment should adopt more secure protocols, such as OAuth 2.0 or Substitutable Medical Applications, Reusable Technologies (SMART) on FHIR [46,47]. Additionally, future work should address patient data privacy [48]. Therefore, strategies such as government policies [49], data anonymization, and pseudonymization should be implemented in future studies to ensure the confidentiality and safety of clinical information [50-52].

Beyond technical privacy, broader deployment of this dashboard would require clear interoperability governance to define how disaster health data are shared across institutions. In Indonesia, disaster-response data flows involve multiple authorities, including BNPB or BPBD at the disaster-coordination level and the MoH HEOC at the health-response level, each operating under distinct regulatory mandates [25,26]. However, to our knowledge, SATUSEHAT does not currently provide a dedicated governance framework for disaster-specific data sharing, separate from routine health data exchange. Establishing data-sharing agreements and access-control policies among disaster-response stakeholders, including defining which entities may access, aggregate, or redistribute FHIR-based patient data during a disaster, remains an open governance gap outside the scope of this technical implementation but is essential for real-world adoption of the dashboard application.

#### Coding and SNOMED CT

The health event classification used in the EMT MDS differs from that used in the Synthea datasets, which use SNOMED CT. As the SATUSEHAT platform also uses SNOMED CT, we chose Synthea to ensure compatibility and attempted to map the SNOMED CT codes to the EMT MDS classifications. However, limited access to the complete SNOMED CT hierarchy restricted our ability to fully map the conditions in the dataset to the EMT MDS classifications.

### Conclusions

This study demonstrated that data collected using FHIR can be effectively displayed through a dashboard application. The data displayed can facilitate communication between the medical team and the command center. Additionally, various stakeholders, including government agencies such as the MoH and disaster management agencies such as the BNPB, may support the decision-making process. Although this application was designed to be implementable on the SATUSEHAT platform and to handle real-world data, further external evaluations involving various stakeholders in disaster management in Indonesia will be required in the future.

## Abbreviations

BNPB: *Badan Nasional Penanggulangan Bencana*
BPBD: *Badan Penanggulangan Bencana Daerah*
DHIS2: District Health Information Software 2
EMT: emergency medical team
EMT MDS: Emergency Medical Teams Minimum Dataset
EMTCC: Emergency Medical Team Coordination Cell
FHIR: Fast Healthcare Interoperability Resources
HAPI: HL7 Application Programming Interface
HEOC: Health Emergency Operation Center
HL7: Health Level 7
LLM: large language model
MoH: Ministry of Health
RESTful: Representational State Transfer
SIPKK: *Sistem Penilaian Kapabilitas Kelembagaan*
SMART: Substitutable Medical Applications, Reusable Technologies
SNOMED CT: Systematized Nomenclature of Medicine–Clinical Terms
WHO: World Health Organization
